# Exposure to Biomass Smoke Extract Enhances Fibronectin Release from Fibroblasts

**DOI:** 10.1371/journal.pone.0083938

**Published:** 2013-12-26

**Authors:** David Krimmer, Yukikazu Ichimaru, Janette Burgess, Judith Black, Brian Oliver

**Affiliations:** 1 The Woolcock Institute of Medical Research, Sydney, Australia; 2 The Discipline of Pharmacology, University of Sydney, Sydney, Australia; University of Giessen Lung Center, Germany

## Abstract

COPD induced following biomass smoke exposure has been reported to be associated with a more fibrotic phenotype than cigarette smoke induced COPD. This study aimed to investigate if biomass smoke induced extracellular matrix (ECM) protein production from primary human lung fibroblasts *in vitro*. Primary human lung fibroblasts (n = 5–10) were stimulated *in vitro* for up to 72 hours with increasing concentrations of biomass smoke extract (BME) or cigarette smoke extract (CSE) prior to being assessed for deposition of ECM proteins, cytokine release, and activation of intracellular signalling molecules. Deposition of the ECM proteins perlecan and fibronectin was upregulated by both CSE (p<0.05) and BME (p<0.05). The release of the neutrophilic chemokine IL-8 was also enhanced by BME. ERK1/2 phosphorylation was significantly upregulated by BME (p<0.05). Chemical inhibition of ERK signalling molecules partially attenuated these effects (p<0.05). Stimulation with endotoxin had no effect. This study demonstrated that BME had similar effects to CSE *in vitro* and had the capacity to directly induce fibrosis by upregulating production of ECM proteins. The mechanisms by which both biomass and cigarette smoke exposure cause lung damage may be similar.

## Introduction

Chronic obstructive pulmonary disease (COPD) is a leading cause of death worldwide, most commonly caused in developed countries by cigarette smoking. Although the link between cigarette smoking and COPD is well founded, epidemiological studies have demonstrated that a substantial proportion of patients with COPD worldwide are never smokers. [1] A growing body of evidence has demonstrated that exposure to smoke from the burning of biomass fuels may be a critical risk factor for the development of COPD in non smokers. [2]

Biomass fuels such as firewood, animal manure and coal are commonly used for heating and cooking around the world. It is estimated that 3 billion people are exposed to indoor smoke from the burning of biomass fuels. [3] Women who cook with biomass fuels are more likely to report respiratory symptoms of cough and wheeze, and have poorer lung function compared to women who do not use biomass fuels for cooking. [4]

Biomass smoke exposure has a similar association with the development of COPD as cigarette smoking, [5] with biomass exposure estimated to increase the risk of developing COPD by 2.4 times. [6] Pathological features of biomass smoke induced COPD include bronchial anthracofibrosis, [7] small airway disease [8] and chronic bronchitis. [5] Biomass exposure can lead to both restrictive and obstructive effects on breathing, with the most commonly reported change in lung function in those exposed to biomass exposure being a decline in forced expiratory volume in 1-second (FEV_1_). [1], [5], [8]

Extensive imaging [9], [10] and histological [11] studies have demonstrated that thickening of the small airway walls is the major contributing factor in COPD to the decline in FEV_1_. In COPD, thickening of the airway wall is characterised by increased fibrotic deposition of extracellular matrix (ECM) proteins, [12] vascularisation [13] and thickening of the epithelial layer. [11] Thickened airways have been observed during autopsies of subjects with significant biomass smoke exposure, where significant airway fibrosis was observed in both the large and the small airways and the extent of fibrosis exceeded that of those of cigarette smokers. [14] Therefore, the decline in FEV_1_ associated with biomass smoke exposure [1], [5], [8] may be due to biomass smoke exposure causing airway thickening.

Biomass smoke is composed of over 200 different compounds, many of which can be inhaled into the small airways. [3] It contains particulate matter, carbon monoxide, polyaromatic hydrocarbons, free radicals, high levels of endotoxin, [15] and many other volatile organic compounds. [16] Although biomass smoke exposure is a considerable risk factor for the development of COPD in non smokers, very little research has been undertaken to determine the mechanisms by which biomass smoke exposure leads to detrimental changes in lung function.

This study aimed to investigate the effect of biomass smoke exposure on human lung cells *in vitro*, specifically examining biomass smoke on markers of airway remodelling, such as deposition of ECM proteins and cytokine release, to demonstrate that biomass smoke exposure can directly cause changes that may relate to airway remodelling, and thus the decline in FEV_1_ *in vivo*.

## Methods

### Ethics statement

Human airway tissue was obtained from explanted and resected lungs and post mortem organ donors with ethical approval from The University of Sydney and participating hospitals (Concord Repatriation General Hospital, Sydney South West Area Health Service and Royal Price Alfred Hospital) for sample collection. All volunteers, or their next of kin, provided written informed consent.

### Chemicals

The following chemicals were obtained from the companies indicated:

DMEM, dimethyl sulfoxide (DMSO), BSA, ammonium hydroxide, Direct red 80, Picric Acid (Sigma, St Louis, MI), PBS, penicillin, streptomycin, amphotericin B (Invitrogen, Carslbad, CA), FBS (Bovogen, East Keilor, Australia), UO126, PD98059 (Calbiochem, San Diego, CA).

### Fibroblast isolation

Approval for all experiments with human lung was provided by the Human Ethics Committees of The University of Sydney and the Sydney South West Area Health Service. Human lung fibroblasts were isolated from lung tissue obtained from donors undergoing resection for thoracic malignancies or lung transplantation and they gave written, informed consent. Donor characteristics, where available, were obtained with permission, from the patient's medical records post-surgery. Disease diagnosis was made by a physician according to current guidelines. We were unable to obtain data on donor's exposure to environmental pollution or biomass smoke prior to sample collection. Characteristics of the donors, including age, smoking status, pack years and lung function, are provided in table 1. Human lung fibroblasts were isolated from distal small airways as previously published [17]. To obtain human lung fibroblasts, cells were obtained from proximal lung tissue containing small airways (<1 mm) which were deemed to be free of tumour following pathological examination. This tissue was minced in 1–2 mm pieces into sterile Hanks Buffered Saline Solution (Hanks) and centrifuged for 5 minutes at 1000 rpm. Supernatant was aspirated and the tissue pellet was resuspended and plated onto tissue culture grade plastic flasks in 10% (vol/vol) FBS/2% antibiotics/DMEM.

**Table 1:** Characteristics of fibroblasts donors.

Donor #	Age	Gender	Disease	Surgery	Smoker	FEV_1_ (% predicted)	FVC (% predicted)	FEV_1_:FVC
1	59	F	Carcinoma	R	No	93	79	0.94
2	73	M	NSCLC	R	ex	59	51	0.93
3	45	F	COPD	T	ex	N/A	N/A	N/A
4	60	M	Bronchiectasis	T	N/A	22	64	0.51
5	54	F	Ca + COPD	R	ex	73	93	0.63
6	59	M	COPD	T	ex	11	52	0.17
7	69	M	NSCCa + COPD	R	ex	56	59	0.75
8	67	M	NSCLC	R	ex	103	101	0.79
9	71	M	NSCLC	R	No	93	89	0.79
10	72	M	NSCLC	R	ex	83	89	0.73
11	60	F	NSCLC	R	ex	99	107	0.79
12	60	M	IPF	T	N/A	N/A	N/A	N/A
13	74	F	NSCLC	R	N/A	N/A	N/A	N/A
14	53	M	Mass + Pneumonia	R	N/A	N/A	N/A	N/A
15	75	M	NSCLC	R	N/A	N/A	N/A	N/A
16	64	M	Carcinoma	R	ex	93	95	0.97
17	57	M	Sarcoidosis	R	N/A	56	60	0.72
18	73	M	NSCLC + COPD	R	ex	67	73	0.73
19	69	F	NSCLC	R	N/A	N/A	N/A	N/A
20	74	F	COPD	T	ex	64	55	0.93
21	63	M	COPD	T	ex	N/A	N/A	N/A
22	72	F	NSCLC	R	N/A	N/A	N/A	N/A
23	61	M	COPD	T	ex	13	47	0.23
24	43	F	COPD	T	ex	N/A	N/A	N/A
25	60	F	Carcinoma	R	ex	101	92	0.86
26	56	M	COPD	T	ex	14	35	0.31

Lung function data presented as % of predicted values for donor's gender, age and height. F: Female, M: Male, NSCLC: non small cell lung carcinoma, COPD: chronic obstructive pulmonary disease, Ca: carcinoma, IPF: idiopathic pulmonary fibrosis, R: resection, T: Transplant. FEV_1_ forced expiratory volume in 1 second. FVC: forced vital capacity. N/A: data not available.

The lung fibroblasts proceeded to grow out of the tissue fragments to form a monolayer covering of the tissue culture flasks. Once the monolayer of cells was confluent, the cells were passaged. All experiments were carried out using cells between passage 3 and 6.

In preparation for *in vitro* experimentation, cells were seeded in 96 &/or 12 well plates for 72 hours in 5% (vol/vol) FBS/antibiotics/DMEM at a density of 1×10^4^ cells/cm^2^. Cells were equilibrated before experimental stimulation for 24 hours in 0.1% (vol/vol) FBS/antibiotics/DMEM.

### Cell culture

Human lung fibroblasts were seeded at a density of 3.2×10^4^ cells/cm^2^ in 5% FBS/antibiotics/DMEM for 72 hours. Cells were then equilibrated by incubation in 0.1% FBS/antibiotics/DMEM for 24 hours prior to stimulation.

### Biomass smoke extract preparation

Biomass smoke extract (BME) was prepared fresh by combusting 500 mg of biomass (*Quercus robur* (*English Oak))* and bubbling through 25 ml DMEM. This solution, 100% BME, was then diluted in 0.1% (vol/vol) FBS/antibiotic/DMEM and applied to cells within 30 minutes of preparation.

Fibroblasts were incubated with 1%, 5%, 10% and 20% BME in 0.1% FBS/antibiotics/DMEM for 72 hours before supernatants were collected and cell deposited ECM was exposed. The ECM was exposed by first washing the cells in PBS, before cells were lysed by exposure to 0.1 M NH_4_OH (Worsley Alumina, WA, Australia) for 15 minutes. Plates were then washed three additional times in PBS to remove cell debris, as previously described. [17] Smoke exposed and smoke naïve cells were cultured in separate, isolated incubators to prevent smoke extract ‘leaching’ across into naïve cells.

### Cigarette smoke extract preparation

Cigarette smoke extract (CSE) was prepared as previously described. [17] Briefly, the smoke from one commercial, high-tar cigarette was bubbled through 25 ml DMEM to make a 100% CSE solution.

### Particle concentration analysis

Analysis of the relative quantities of particulate matter in the smoke generated by burning biomass and cigarettes were assessed using a Lasair II laser particle counter (Particle Measurement Systems, Boulder, CO) which samples particle sizes in the ranges of 0.3–0.5, 0.5–1, 1.0–5, 5–10, 10–25 and >25 µm. One litre of freshly generated smoke was diluted into 39 litres of medical grade N_2_ gas, of which 28.31 litres was sampled to give a total particle count from which values of particles per m^3^ were calculated. Experiments were performed in triplicate.

### Assessment of endotoxin levels and pH

Relative levels of endotoxin in BME and CSE were assessed using a commercially available *limulus* amebocyte lysate (LAL) assay according to the manufacturer's instructions (Cape Cod, East Falmouth, MA). Briefly, the quantity of lipopolysaccharide (LPS) contained in BME and CSE was measured in duplicate using 1∶10, 1∶100 and 1∶1000 dilutions of each sample made with pyrogen-free water using a chromogenic LAL assay with β-glucan inhibiting buffer and a standard curve with a sensitivity range of 0.008 - 2 EU/ml. To evaluate possible sample interference with LPS measurements, additional duplicates of each sample were spiked with LPS added directly to the assay well. Total LPS bioactivity was measured by kinetic assay using a VersaMax microplate reader with SoftMax Pro 5 software.

The pH of samples was measured electronically using a calibrated Cyberscan 500 pH probe (ThermoFisher Scientific).

### Growth factor stimulation

In addition to BME and CSE exposure, in some experiments cells were stimulated with 1 ng/ml recombinant human TGF-β_1_ (R&D Systems, Minneapolis, MN) or 0.05, 0.5 and 5 µg/mL purified LPS, a major component in endotoxin, as control stimuli.

### Cytotoxicity assay

Cytotoxicity was assessed by trypan blue exclusion, a commercially available lactate dehydrogenase (LDH) assay and a commercially available 3-(4,5-Dimethylthiazol-2-yl)-2,5-Diphenyltetrazolium Bromide (MTT) assay according to the manufacturer's instructions (Sigma).

### Extracellular matrix ELISA

Relative levels of fibronectin and perlecan deposited into the ECM were assessed by an ECM ELISA. Briefly, cells were washed in PBS, and then lysed using 0.1 M NH_4_OH (Worsley Alumina, WA, Australia) for 15 minutes. Plates were then washed three additional times to remove cell debris. Deposition of proteins into the ECM was measured using 4 µg/ml mouse-anti fibronectin C-terminal (Chemicon, Billerica, MA) antibodies and 2 µg/ml mouse anti-perlecan antibodies in 1% BSA/PBS as previously published.[17]

### Signalling pathways - Analysis of ERK activation by western blotting

To assess the activation of intracellular signalling molecules in fibroblasts following stimulation with BME or CSE, relative levels of extracellular regulated kinase (ERK)/1 and ERK/2 phosphorylation from cell lysates collected after 10, 20, and 30 minutes stimulation with BME or CSE was assessed by western blotting. Briefly,lysates were diluted in a 5x SDS-PAGE sample buffer, denatured, separated by polyacrylamide (10%) gel electrophoresis and then transferred onto a polyvinylidene difluoride (PVDF) membrane (Millipore). Following protein transfer, non specific binding was blocked via incubation of the membrane for 1 hour with 5% BSA (w/v). Mouse anti-phospho-ERK/1 and phospho-ERK/2 antibodies (Cell signalling) were used to identify phospho-ERK/1 and phospho-ERK/2 respectively, which were detected using luminescence via secondary HRP conjugated anti-mouse antibodies (Dako, Glostrup, Denmark) and SuperSignal luminescence buffer (Gibco), using a Kodak Image Station Camera and software. The membranes were then stripped using stripping buffer (0.063 mM Tris, pH 6.8, 2% SDS, 0.7% β-mercaptoethanol) and reincubated with 0.002 ng/ml mouse anti-glyceraldehyde-3-phosphate dehydrogenase (GAPDH) monoclonal antibody (Chemicon, Millipore, Temecula, CA) after blocking.

### ECM ELISA

Fibroblasts were treated with or without the ERK MAPK inhibitors UO126 or PD98059 in appropriate concentrations of DMSO in 0.1% FBS/antibiotics/DMEM. After one hour, media was aspirated before the addition of 1%, 5%, 10% and 20% BME or 5% CSE in the presence of inhibitors for 72 hours. Following stimulation, relative levels of fibronectin and perlecan deposited into the ECM were assessed by an ECM ELISA and levels of IL-8 released into the supernatant were assessed by ELISA.

### IL-8 ELISA

Levels of IL-8 released into the supernatant following stimulation with 1%, 5%, 10% and 20% BME for 72 hours were assessed using commercial antibody kits according to the manufacturer's instructions (R&D Systems).

### Data analysis

ECM protein deposition was corrected by subtracting the absorbance reading of “no-cell” negative control wells from the fibroblast containing wells to remove background absorbance. All data were collated using Microsoft Excel Software and analysed using GraphPad Prism 5.0 (GraphPad, La Jolla, CA). Differences were considered to be significant when p<0.05.

## Results

### Analysis of BME and CSE

The mean endotoxin level in CSE was 4.23±2.08 EU/ml, whilst BME contained 2.60±0.59 EU/ml, and these values where not significantly different. The pH of 100% BME (7.84±0.01) was slightly higher than 100% CSE (7.57±0.01) and 0.1% FBS/antibiotics/DMEM (7.57±0.03), indicating a slightly more alkaline solution. However, when the BME was diluted in 0.1% FBS/antibiotics/DMEM to the concentrations used in the experiments, the pH of 20% BME was 7.59 (±0.04) and 10% BME was 7.56 (±0.02).

**Figure 1:**
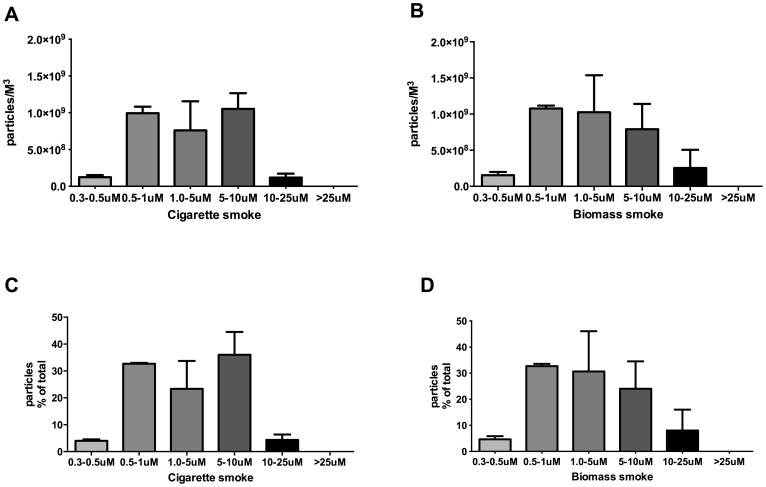
Biomass smoke and cigarette smoke have similar particle consistency. Quantity and distribution of particles from the smoke of one cigarette (A) or one unit of biomass (B) as measured by laser particle counter. Data expressed as particles per cubic metre or as % of total particle counts (C–D) for particles in the size ranges of 0.3–0.5, 0.5–1, 1–5, 5–10, 10–25 or <25 uM diameter. Bars represent mean ± SEM. Each experiment was performed in triplicate.

Both biomass smoke and cigarette smoke contained very high levels of small particles,with biomass smoke containing an average of 3.05×10^9^ particles/m^3^ in the 0.3–10 µm size range and cigarette smoke containing an average of 2.93×10^9^ particles/m^3^ of the same size range (Figure 1 A, B). Biomass and mainstream cigarette smoke contained similar proportions of small particles, respectively yielding 92.0% and 95.6% of total particles in the 0.3–10 µm size range (Figure 1C, D).

### High concentrations of biomass extract are cytotoxic

**Figure 2:**
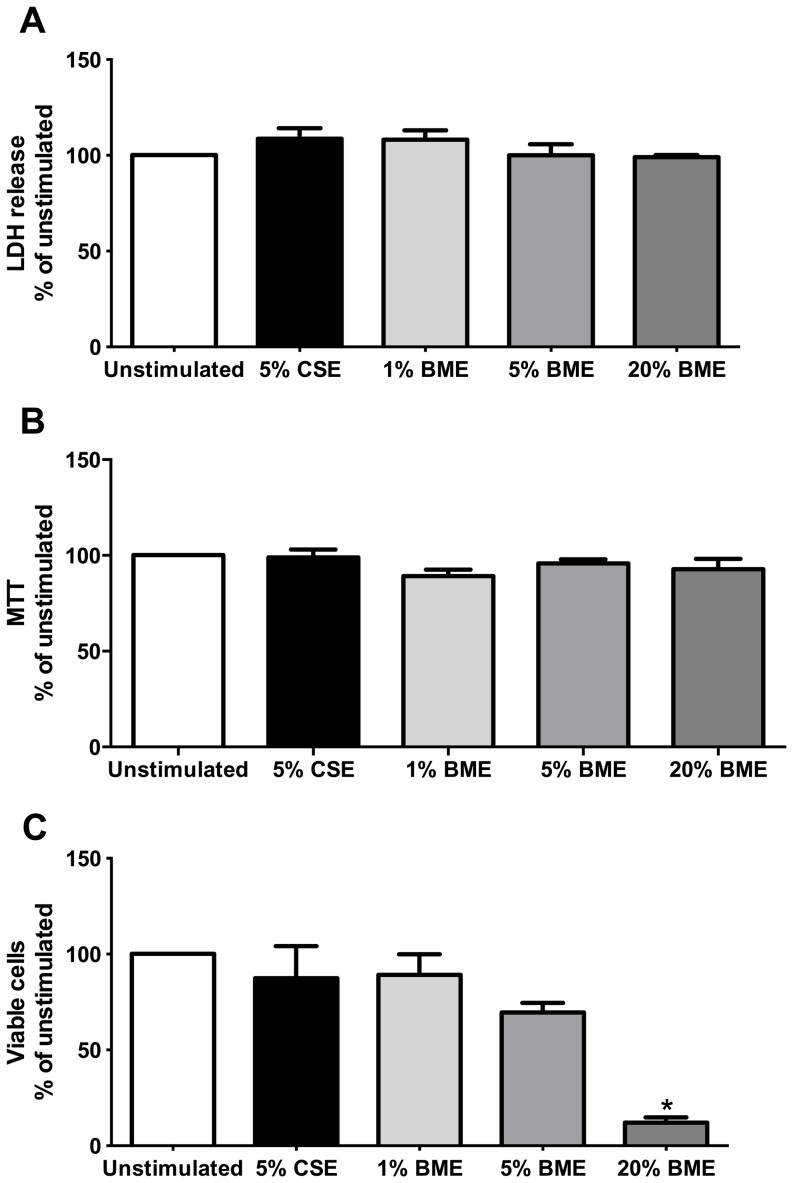
High concentrations of biomass smoke are cytotoxic. Assessment of cytotoxicity of biomass smoke extract (BME) to human lung fibroblasts (n = 3) as measured by LDH assay (A), MTT assay (B) or manual cell counts (C) following stimulation with 5% cigarette smoke extract (CSE) or 1, 5, or 20% BME in 0.1% FBS/DMEM. Data expressed as % of unstimulated. Bars represent mean ± SEM. Data analysed by one-way ANOVA with Dunnet's post test.*p<0.05 vs unstimulated, n = 6.

We initially assessed cytotoxicity of 1, 5, 10 and 20% BME via the LDH assay (Figure 2A). The data suggested that BME was not cytotoxic to fibroblasts (n = 3) following 72 hours stimulation. As we had previously observed that high concentrations of CSE were cytotoxic [17], this was an unexpected result. We then assessed the effect of BME on cell viability using a MTT assay (Figure 2B). The data also suggested that BME did not have a significant effect on cell viability (n = 3). We then performed manual cell counts after 72 hours stimulation and these data demonstrated that the stimulation with 1, 5 and 10% BME did not significantly alter the total cell number (Figure 2C), however the number of viable cells present after 72 hours stimulation with 20% BME was significantly decreased compared to controls (p<0.05, n = 6).

### Biomass extract enhances deposition of fibronectin

**Figure 3:**
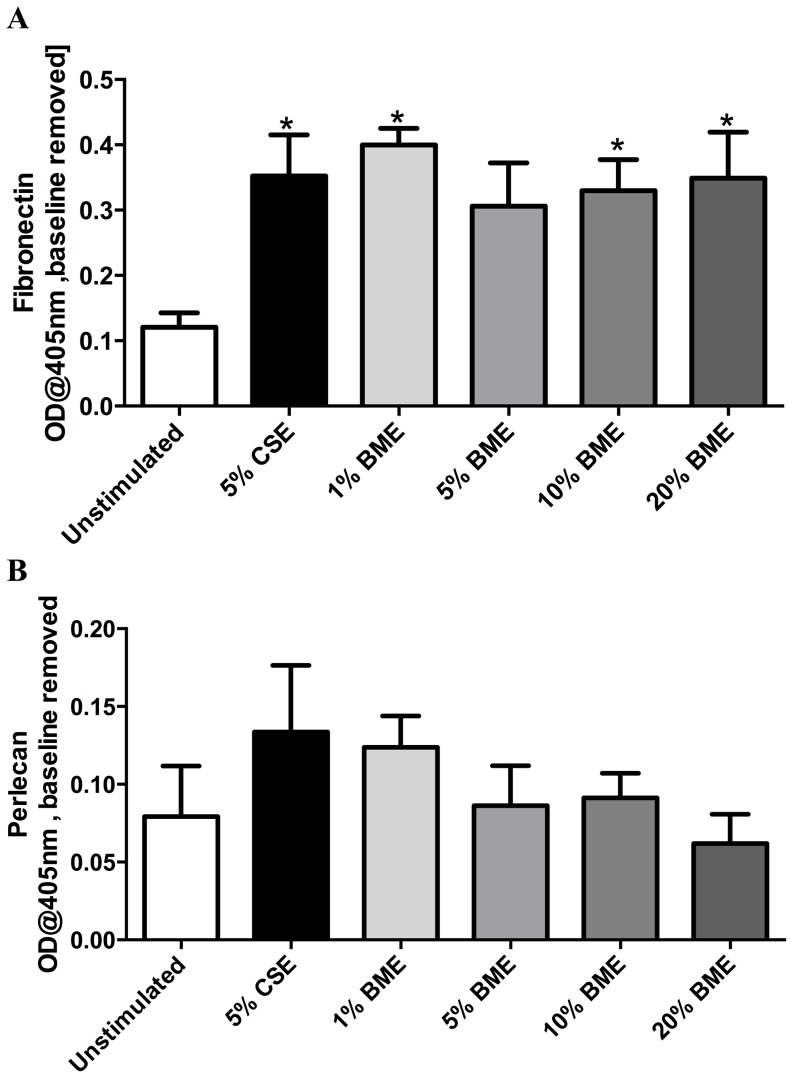
Biomass smoke extract enhances deposition of fibronectin from fibroblasts. Deposition of fibronectin (n = 16) (A) or perlecan (n = 6) (B) from human lung fibroblasts as measured by ECM ELISA following 72 hours stimulation with 5% cigarette smoke extract (CSE) or 1, 5, 10 or 20% biomass smoke extract (BME) in 0.1% FBS/DMEM. Data expressed as absorbance at 405 nm with media alone baseline removed (A,B). Bars represent mean ± SEM. Data analysed by one-way ANOVA with Dunnet's post-test.*p<0.05 vs unstimulated, n = 6–16.

As fibronectin is a significant component of the ECM [18] and is upregulated in COPD, [12] we assessed the deposition of fibronectin from human lung fibroblasts using an ECM ELISA. We found that fibronectin deposition was significantly increased following 72 hours stimulation with 5% CSE, 10% and 20% BME (p<0.05, n = 16) (Figure 3A).

Deposition of the glycosaminoglycan, perlecan, was not altered by stimulation with BME (n = 6)(Figure 3B).

### Chemical inhibitors of ERK MAPK attenuate biomass-induced fibronectin deposition

**Figure 4:**
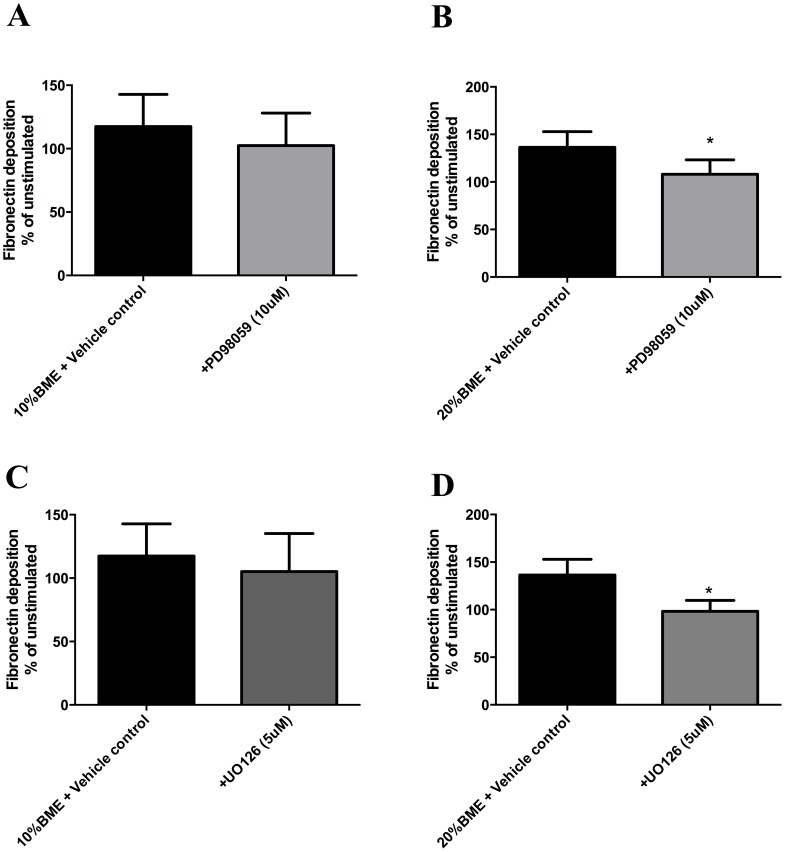
Chemical inhibition of the ERK signalling pathway attenuates biomass smoke induced fibronectin deposition. Fibroblasts were pretreated for 1(10 µM) (A,B) or UO126 (5 µM) (C,D) in DMSO (vehicle control) before stimulation with 10% (A,C) and 20% BME (B,D) in the presence of inhibitors for 72 hours, prior to analysis of fibronectin deposition by ECM ELISA. Data expressed as % of unstimulated. Bars represent mean ± SEM. Data analysed by T-Test. *p<0.05 vs vehicle control, n = 5.

The deposition of fibronectin induced by 20% BME was significantly attenuated following 72 hours stimulation in the presence of both ERK inhibitors PD98059 and UO126 (p<0.05 vs vehicle control, n = 5) (Figure 4B and 4D).

### Biomass extract enhances release of IL-8

As neutrophilic inflammation is a characteristic feature of COPD, we sought to assess the effect of BME on release of the chemotactic cytokine IL-8 by human lung fibroblasts.

**Figure 5:**
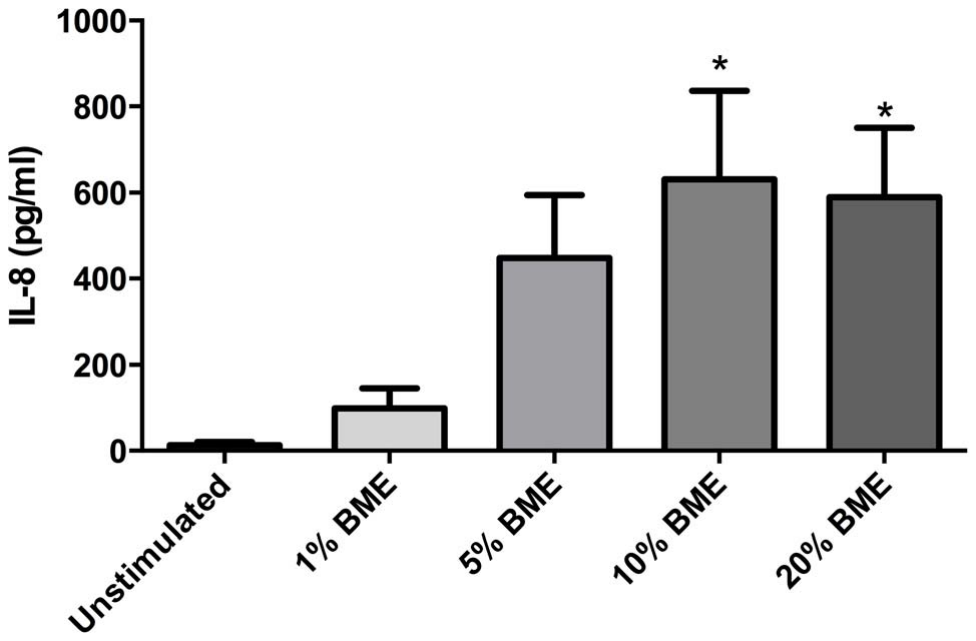
Biomass smoke exposure upregulates release of IL-8 from fibroblasts. Release of interleukin (IL)-8 from human lung fibroblasts (n = 10) in response to 72 hours stimulation with 1, 5, 10 or 20% biomass smoke extract (BME) in 0.1% FBS/DMEM as measured by IL-8 ELISA. Data expressed as pg/ml. Bars represent mean ± SEM. Data analysed by one-way ANOVA with Dunnet's post-test. *p<0.05 vs unstimulated, n = 10.

**Figure 6:**
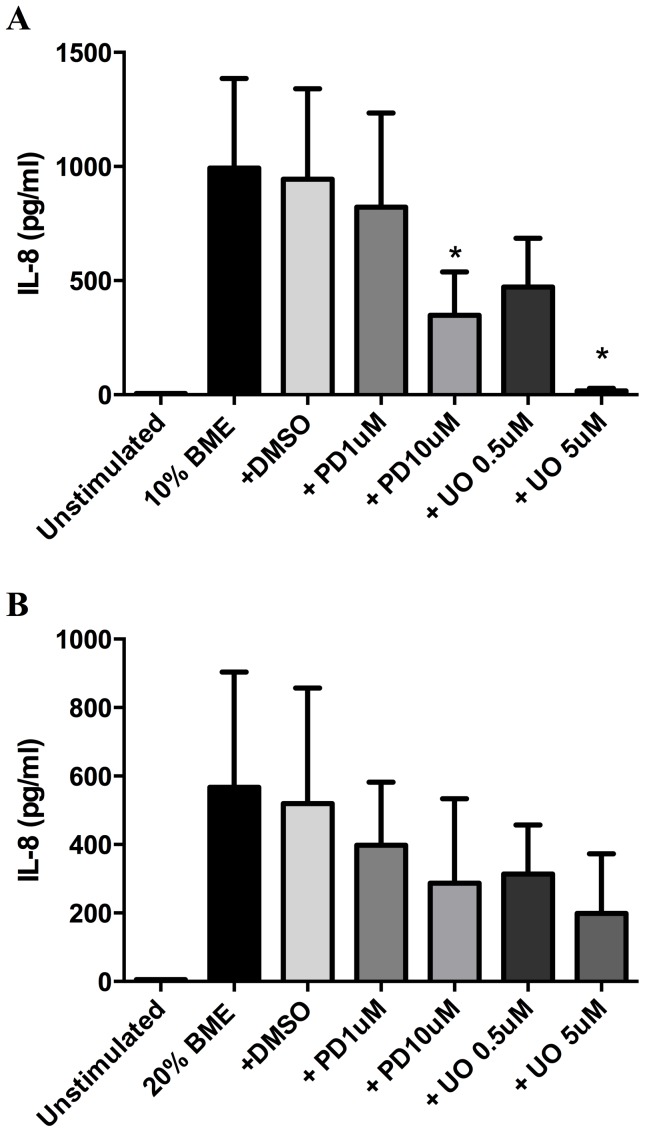
Chemical inhibition of the ERK signalling pathway attenuates BME induced IL-8 release. Fibroblasts were pretreated for 1(1, 10 µM) or UO126 (0.5, 5 µM) in DMSO (vehicle control) before stimulation with 10% (A) and 20% (B) BME in the presence of inhibitors for 72 hours, prior to analysis of IL-8 release by ELISA. Data expressed as pg/ml. Bars represent mean ± SEM. Data analysed by one-way ANOVA with Bonferroni's post-test.*p<0.05 vs vehicle control, n = 5.

Our data demonstrated that after 72 hours stimulation, 10% and 20% BME significantly upregulated the release of IL-8 into the supernatant compared to control (p<0.05, n = 10) (Figure 5). In cells stimulated in the presence of the ERK inhibitors PD98059 (10 µM, 1 µM) and UO126 (5 µM, 0.5 µM), the release of IL-8 induced by 10% BME, but not 20% BME, was significantly attenuated by the higher concentrations of PD98059 and UO126 (p<0.05, n = 5) (Figure 6).

### Biomass extract enhances phosphorylation of ERK

**Figure 7:**
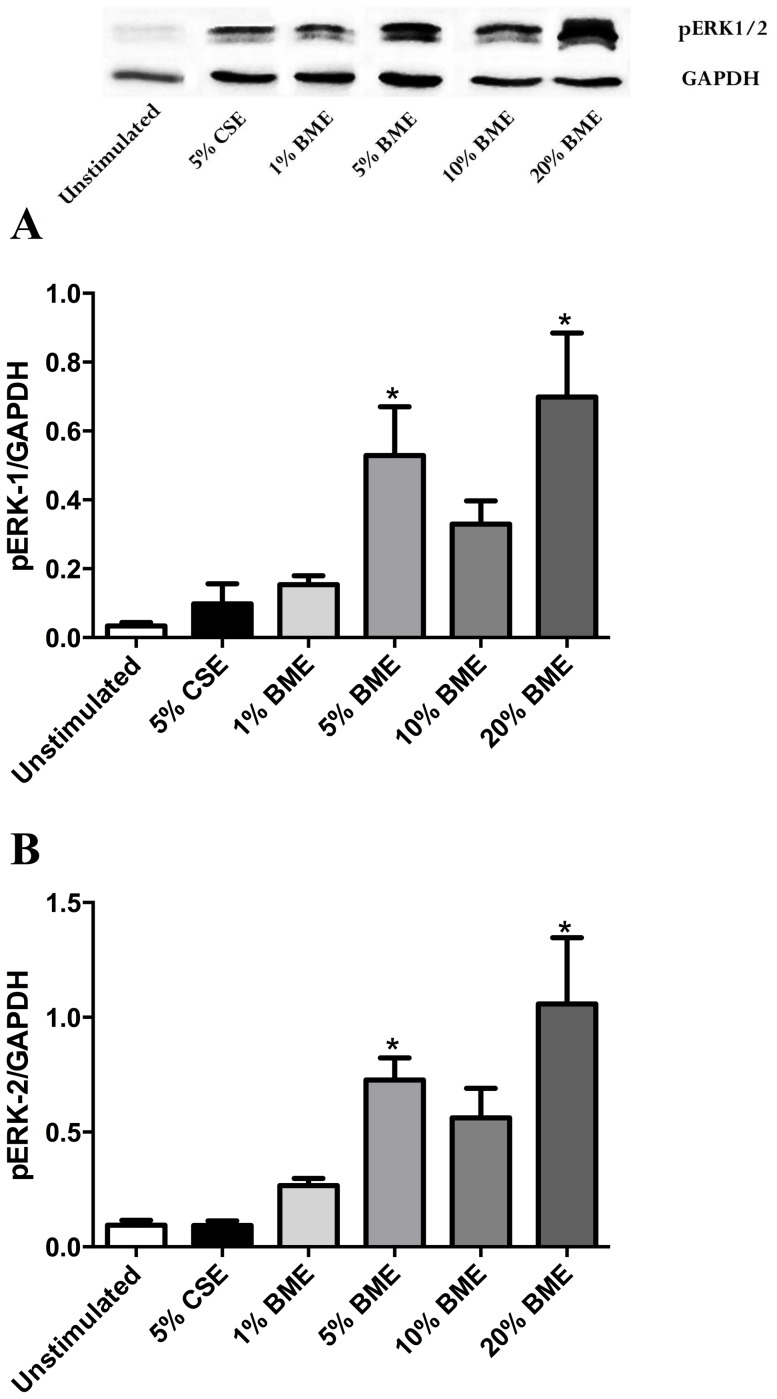
BME activates ERK1/2 signalling molecules. Fibroblasts were stimulated for 2% CSE or 1, 5, 10 or 20% BME in 0.1% FBS/DMEM, before whole cell lysates were collected and ERK1 (A) or ERK/2 (B) phosphorylation was assessed by western blotting. Data expressed as the ratio of pERK over GAPDH (housekeeping protein). Bars represent mean ± SEM. Data analysed by one-way ANOVA with Dunnet's post-test *p<0.05 vs unstimulated, n = 5. Image at top of graph is a representative composite western blot.

As the ERK MAPK signalling pathway has been previously demonstrated to be activated by particulate matter [19] we assessed the phosphorylation of ERK1/2 by BME in human lung fibroblasts via western blotting. Compared to time 0, BME began to increase pERK1/2 after 30 minutes in a dose-related fashion. Following two hours stimulation, pERK1/2 was significantly increased, compared to control, by 5% and 20% BME (p<0.05, n = 6) (Figure 7). Whilst pERK1 levels decreased by 24 hours, pERK2 remained significantly increased by 10% and 20% BME (p<0.05, n = 6) (data not shown).

### LPS does not increase fibronectin deposition or IL-8 release from human lung fibroblasts

**Figure 8:**
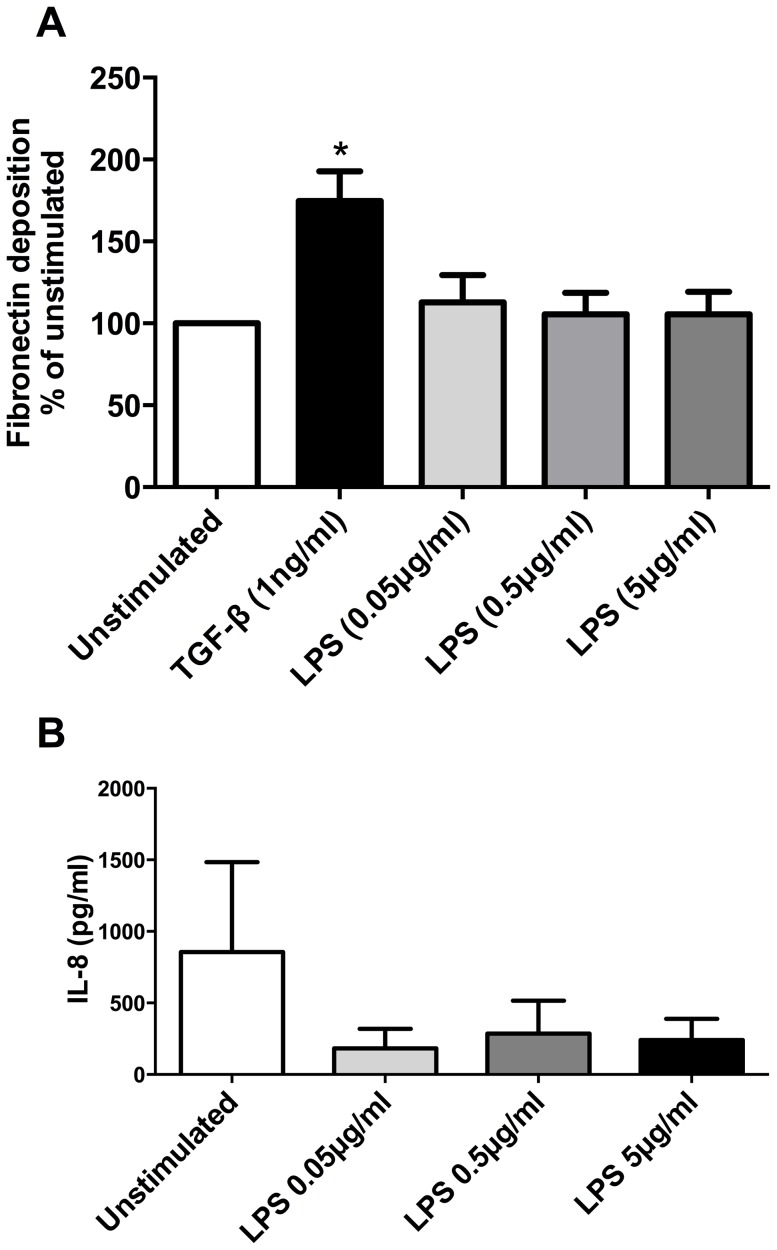
LPS does not induce fibronectin deposition (A) or IL-8 release (B). Human lung fibroblasts were stimulated for 72 µg/ml lipopolysaccharide (LPS) prior to analysis of fibronectin deposition by ECM ELISA (A) or IL-8 release by ELISA (B). The profibrotic cytokine TGF-β_1_ (1 ng/ml) was used as a positive control for fibronectin deposition. Data expressed as % of unstimulated (A) or pg/ml (B). Bars represent mean ± SEM. Data analysed by one-way ANOVA with Dunnet's post-test.*p<0.05 vs unstimulated, n = 5.

As LPS is a major component of endotoxin, which we found to be present in BME, we tested the direct effects of LPS on fibronectin deposition or the release of IL-8 from human lung fibroblasts. The profibrotic cytokine TGF-β_1_ (1 ng/ml), used as a positive control, increased fibronectin deposition as measured by ECM ELISA (p<0.05, n = 5) (Figure 8A). In comparison, stimulation for 72 hours with 0.05, 0.5 and 5 µg/ml LPS did not alter the deposition of fibronectin from human lung fibroblasts.

## Discussion

This study has demonstrated that biomass smoke extract can directly upregulate the deposition of the ECM protein fibronectin and release of the neutrophil attractant chemokine IL-8 from human lung fibroblasts, through a process which may involve activation of the ERK signalling pathway.

As increased matrix deposition is a characteristic of airway remodelling, the pathological changes observed in biomass smoke induced COPD *in vivo* may be, in part, due to direct upregulation of matrix proteins. Thus the decline in FEV_1_ observed in those exposed to biomass smoke may be due to airway remodelling and inflammation which has occurred as a result of biomass smoke exposure.

We found that BME activates the ERK signalling pathway and that the release of IL-8 and deposition of fibronectin were partially attenuated by chemical inhibition of this pathway. ERK has been shown to be activated by CSE in human lung fibroblasts, [20] airway epithelial cells [21] and immune cells. [22] In studies of COPD, ERK can be activated by the nicotine contained in cigarette smoke [23] and ERK is linked to inflammation, [24], [25] enhanced airway smooth muscle proliferation [26] and mucin production. [27]

Whilst chemically inhibiting the ERK pathway did not completely attenuate the effects of BME, we and others have demonstrated the involvement of nuclear factor kappa-B (NF-κB), janus regulated kinase (JNK) and Smad signalling pathways in fibronectin deposition and IL-8 release. [17], [28]–[32] Thus it is reasonable to conclude that whilst ERK activation plays a significant role in BME induced fibronectin deposition and IL-8 release, other signalling molecules are likely to be involved.

Fibronectin is an important component of the ECM that has been demonstrated to play an active role in the pathogenesis of lung disease. Enhanced expression of fibronectin has been observed in COPD [12] and in the bronchial alveolar lavage fluid from smokers. [33] In addition fibroblasts from patients with idiopathic pulmonary fibrosis [34] and COPD [17] produce more fibronectin than controls, suggesting a role of this molecule in fibrosis. We have previously demonstrated that CSE can directly upregulate fibronectin deposition from fibroblasts, [17] whilst others have shown that fibronectin is enhanced by nicotine, [28], [35] ethanol, [36] TGF-β_1_, [37] oxidative stress [38] and mechanical strain. [39] Fibronectin is critically involved in wound repair processes in the lung, and enhanced levels of fibronectin may promote fibrosis. Reduced fibronectin levels can directly diminish the rate of wound closure of airway epithelial cells [40] and fibronectin knock-out mice fail to develop fibrosis in response to bleomycin. [41] Fibronectin directly mediates and enhances migration of small airway and alveolar epithelial cells, [42], [43] and also enhances proliferation of lung carcinoma cells [44] and airway smooth muscle cells. [45]–[47] Whilst there have not been detailed immunohistochemical analyses of airways of persons who develop biomass smoke induced COPD, we demonstrate *in vitro* that BME has the capacity to enhance the deposition of fibronectin. Future studies are warranted to determine if this observation correlates to the pathology of airway remodelling *in vivo*.

Neutrophilic inflammation is a hallmark characteristic of COPD. [48] Interleukin 8 is the main chemotactic mediator for neutrophils, having been established as the key mediator driving neutrophilic inflammation *in vitro* and *in vivo*. [49], [50] IL-8 is of interest in COPD as patients with COPD have more IL-8 in both sputum and serum than asthmatics or healthy controls. [51] Concurrently, they also have greater numbers of neutrophils in sputum. [52] In patients with COPD, a significant inverse correlation has been observed between levels of IL-8 in the epithelial layer and FEV_1_. [53] Thus by enhancing the release of IL-8, biomass smoke may mediate the recruitment of neutrophils which in turn can release inflammatory mediators and proteolytic enzymes, thus having an active role in the progression of obstructive lung disease.

The deposition of perlecan was not upregulated by BME in this study. We previously demonstrated that the deposition of fibronectin and perlecan following CSE exposure involved different signalling pathways. [17] Specifically CSE induced fibronectin involved the NF-κB pathway, whilst CSE induced perlecan involved the activation of the Janus Kinase (JAK)/Stat pathway. A possible mechanism for the upregulation of fibronectin, but not perlecan, observed in this study may be that BME does not activate the JAK/Stat signalling pathway. A limitation of our study is that we did not examine the activation of this pathway, so further research on the mechanisms by which fibronectin is upregulated is warranted.

Whilst endotoxin is a substantial component of BME, the direct stimulation of human lung fibroblasts with LPS failed to induce similar changes to those observed when cells were directly stimulated with BME. This finding demonstrates that the increase in fibronectin deposition and IL-8 observed are not solely due to the presence of endotoxin.

We were not able to ascertain the molecules present in BME responsible for the observed changes in this study as biomass smoke contains over 200 different compounds. Likely candidates may be polyaromatic hydrocarbons [54] or particulate matter such as PM_2.5_ itself. [55] Particulates <10 uM in diameter may drive the harmful effects of inhaled substances on the respiratory system. [56] This study demonstrated similar particulate profiles between biomass and cigarette smoke, with the majority of particles being in the 0.5–10 uM range for both substances. Therefore, as both biomass and cigarette smoke have similar particulate profiles, it is reasonable to conclude that exposure to biomass smoke may induce similar pathological effects as cigarette smoke, and this may reflect the similarity in particle size of both stimuli.

A recent study demonstrated LPS directly induced IL-8 from human lung fibroblasts after 24 hours, [57] a finding which contrasts our study, whereby LPS did not induce IL-8 release at 72 hours. Whilst the differences may be due to the time points examined and the cell culture conditions, further research on the influence of endotoxin on cellular physiology is warranted.

Biomass smoke exposure has an enormous impact on health in developing areas, contributing greatly to morbidity and mortality. [2] Whilst priority needs to be given to interventions to reduce biomass smoke exposure, further research is also needed to better characterise both the mechanisms and pathology of biomass smoke induced lung disease, so as to enable better therapeutic options for those who have already had substantial exposure.

In conclusion, we have demonstrated that exposure to biomass smoke *in vitro* can enhance the deposition of a key ECM protein and the release of a major neutrophilic chemotactic mediator from human lung fibroblasts, via the activation of the ERK signalling pathway. These findings provide a novel mechanism for lung injury following biomass smoke exposure.
